# Full-Genome Sequence Analysis of a Natural Reassortant H4N2 Avian Influenza Virus Isolated from a Domestic Duck in Southern China

**DOI:** 10.1128/genomeA.00894-14

**Published:** 2014-09-11

**Authors:** Aiqiong Wu, Zhixun Xie, Liji Xie, Zhiqin Xie, Sisi Luo, Xianwen Deng, Li Huang, Jiaoling Huang, Tingting Zeng

**Affiliations:** a College of Animal Science and Technology, Guangxi University, Nanning, Guangxi Province, China; b Guangxi Key Laboratory of Animal Vaccines and Diagnostics, Guangxi Veterinary Research Institute, Nanning, Guangxi Province, China

## Abstract

We report here the complete genome sequence of a novel reassortant H4N2 avian influenza virus strain, A/duck/Guangxi/125D17/2012(H4N2) (GX125D17), isolated from a duck in Guangxi Province, China in 2012. We obtained the complete genome sequence of the GX125D17 virus isolation by PCR, cloning, and sequencing. Sequence analysis revealed that this H4N2 virus strain was a novel reassortant avian inﬂuenza virus (AIV). Information about the complete genome sequence of the GX125D17 virus strain will be useful for epidemiological studies.

## GENOME ANNOUNCEMENT

Avian influenza virus (AIV) is a single-strained, negative-sense RNA virus belonging to the family *Orthomyxoviridae* (1,2). Avian influenza viruses are divided into subtypes based on the two major surface glycoproteins, hemagglutinin (HA), and neuraminidase (NA). So far, 16 HA and 9 NA subtypes have been identified (1, 3–6). The H4 subtype of AIVs have been circulating and evolving in live poultry markets (LPM) in China (7). The H4N2 subtype of avian influenza virus has infected migratory water birds (8) and domestic ducks (9). In addition, the H4 avian influenza virus has infected pigs (10) and poses a threat to mammals. Thus, enhanced the surveillance of H4N2 subtype of AIVs is of great importance.

In this study, an H4N2 strain, named A/Duck/Guangxi/125D17/2012(H4N1), was isolated from a duck in Guangxi, southern China in 2012. The nucleotide sequences of this virus strain were amplified by reverse transcription-PCR (RT-PCR) using universal primers (11–13). The amplified products were purified and cloned into the pMD18-T vector (TaKaRa) and then sequenced (Invitrogen, Shanghai, China). Sequences were assembled and manually edited to generate the final genome sequence (14).

The complete genome of GX125D17 strain consists of PB2, PB1, PA, HA, NP, NA, M, and NS segments. The eight genes encoded the following proteins, followed by the deduced amino acid: PB2, 760 aa; PB1, 757 aa; PA, 716 aa; HA, 564 aa; NP, 498 aa; NA, 469 aa; M1, 252 aa; M2, 97 aa; NS1, 230 aa; and NS2, 121 aa. The amino acid sequence at the cleavage site in the HA molecule is EKASR↓GLF, which is characteristic of low-pathogenicity avian influenza virus. Analysis of potential glycosylation sites of surface proteins revealed five potential N-glycosylation sites in HA (18 to 21, 34 to 37, 178 to 181, 310 to 313, and 497 to 500) and seven potential N-glycosylation sites in NA (61 to 64, 69 to 72, 70 to 73, 146 to 149, 200 to 203, 234 to 237, and 402 to 405).

Sequence analysis revealed that the nucleotide sequences of the HA and NA genes of the GX125D17 strain both belong to the Eurasian lineage. The nucleotide homology comparisons revealed that the HA gene of this strain shares 99% homology with the HA gene of a Korea wild-bird AIV strain, A/wild duck/Korea/CSM4-28/2010(H4N6). The NA and NP genes both share the highest homology (≥98%) with those of the H1N2 isolate A/duck/Guangxi/GXd-1/2011(H1N2). The NS gene shares 97% homology with that of the H3N8 isolate A/duck/Chabarovsk/1610/1972(H3N8). The PB1 gene shares the highest homology (98%) with that of the H4N6 isolate A/wild duck/Korea/SH5-26/2008(H4N6). The PB2 gene shares the highest homology (98%) with that of the H7N7 isolate A/common teal/Hong Kong/MPL634/2011(H7N7). The M gene shares 99% homology with the M gene of the H10N8 isolate, A/duck/Guangdong/E1/2012(H10N8). The PA gene shares 99% homology with the PA gene of H1N2 isolate, A/wild waterfowl/Dongting/C2383/2012(H1N2). In conclusion, the GX125D17 virus isolation was proved to be a novel reassortant AIV.

These data will be helpful in epidemiological studies on H4N2 subtype of AIVs in southern China.

### Nucleotide sequence accession numbers.

The genome sequences of A/Duck/Guangxi/125D17/2012(H4N2) have been deposited in GenBank under accession numbers KJ881013 to KJ881020.
